# Quantification of bone changes in a collagen-induced arthritis mouse model by reconstructed three dimensional micro-CT

**DOI:** 10.1186/1480-9222-15-8

**Published:** 2013-07-15

**Authors:** Shu Yang, Anne M Hollister, Elysse A Orchard, Shubnum I Chaudhery, Dmitry V Ostanin, Stephen J Lokitz, J Michael Mathis

**Affiliations:** 1 Department of Cellular Biology and Anatomy, Louisiana State University Health Sciences Center, 1501 Kings Hwy, Shreveport, LA 71130, USA; 2 Department of Orthopedics, Louisiana State University Health Sciences Center, 1501 Kings Hwy, Shreveport, LA 71130, USA; 3 Department of Animal Resources, Louisiana State University Health Sciences Center, Shreveport, LA 71130, USA; 4 Department of Pharmacology, Toxicology, and Neurosciences, Louisiana State University Health Sciences Center, Shreveport, LA 71130, USA; 5 Department of Pathology, Louisiana State University Health Sciences Center, Shreveport, LA 71130, USA; 6 Department of Medicine, Louisiana State University Health Sciences Center, Shreveport, LA 71130, USA; 7 Department of Radiology, Louisiana State University Health Sciences Center, Shreveport, LA 71130, USA; 8 PET Imaging Center, Biomedical Research Foundation of Northwest Louisiana, Shreveport, LA 71130, USA

**Keywords:** Rheumatoid arthritis, Micro-CT, Computed tomography, Imaging, Collagen induced arthritis, Bone density, Bone volume, Disease index

## Abstract

**Background:**

Inflammatory arthritis is a chronic disease, resulting in synovitis and subchondral and bone area destruction, which can severely affect a patient’s quality of life. The most common form of inflammatory arthritis is rheumatoid arthritis (RA) in which many of the disease mechanisms are not well understood. The collagen-induced arthritis (CIA) mouse model is similar to RA as it exhibits joint space narrowing and bone erosion as well as involves inflammatory factors and cellular players that have been implicated in RA pathogenesis. Quantitative data for disease progression in RA models is difficult to obtain as serum blood markers may not always reflect disease state and physical disease indexes are subjective. Thus, it is important to develop tools to objectively assess disease progression in CIA.

**Results:**

Micro-CT (Computed Tomography) is a relatively mature technology that has been used to track a variety of anatomical changes in small animals. In this study, micro-CT scans of several joints of control and CIA mice were acquired at 0, 4, 7, and 9 weeks after the immunization with collagen type II. Each micro-CT scan was analyzed by applying a segmentation algorithm to individual slices in each image set to provide 3-dimensional representations of specific bones including the humerus, femur, and tibia. From these representations, the volume and mean density of these bones were measured and compared. This analysis showed that both the volume and the density of each measured bone of the CIA mice were significantly smaller than those of the controls at week 7.

**Conclusions:**

This study demonstrates that micro-CT can be used to quantify bone changes in the CIA mouse model as an alternative to disease index assessments. In conclusion, micro-CT could be useful as a non-invasive method to monitor the efficacy of new treatments for RA tested in small animals.

## Background

Inflammatory arthritis is a debilitating progressive disease, affecting approximate 1% of the world population [1,2]. Inflammatory arthritis has two representative characteristics: swelling and joint destruction [3,4]. Rheumatoid arthritis (RA) is the most common form of inflammatory arthritis, consisting of almost 50% of the inflammatory arthritis patients in the world. The incidence of RA ranges from 5 to 50 per 100,000 adults in industrialized countries, accounting for roughly 1.5 million cases in the United States [5,6]. The structural damage in RA is characterized by ligament and soft tissue inflammation, joint instability, cartilage degradation, and bone erosion. Cartilage and bone erosion are both consequences of the inflammatory reactions [7].

Structural damage in RA typically includes cartilage and bone reabsorption. Traditionally, structural damages caused by RA have been assessed using conventional radiography to detect cortical bone erosion, joint space narrowing, and peri-articular osteoporosis [8]. Radiography is also used routinely for diagnosing and monitoring of RA patients in the clinical setting [9,10]. However, conventional radiography is not sensitive enough to assess early joint erosion or inflammatory soft tissue damage and is limited by the two-dimensional visualization which results in projection and plane of section errors [8]. Other non-invasive clinical assessment tools include magnetic resonance imaging (MRI) and ultrasound (US), but these have proven even less sensitive and accurate compared with computed tomography (CT) for detecting bone erosion [10,11]. It has been suggested that micro-CT may yield better results in interpreting and identifying the damaged area in bones of small joints, because micro-CT is reproducible and quantitative [12].

Recent studies have shown that micro-CT is the preferred method for detecting bone erosions in RA [13]. Micro-CT facilitated by automated image analysis can be used to quantify cortical bone loss and is commonly used for small animal models to detect morphological changes [14]. The advantages of micro-CT are good spatial resolution (50 μm), short acquisition times, and that all three planes (transverse, coronal, and sagittal) are acquired simultaneously [15].

The collagen-induced arthritis (CIA) rodent model is widely used for pre-clinical investigations of RA including treatment assessment and prognostic evaluation [16]. Results of Brand *et al*. [17] show that most of the susceptible strains injected with collagen II produced an 80 to 100% incidence of arthritis with onset between 21 and 28 days. CIA models have susceptibility to both the major histocompatibility complex (MHC) class II molecules and the species of type II collagen used for immunization. The two strains of mice most widely for CIA are DBA/1 (H-2^q^) mice and B10 RIII (H-2^r^) [18]. The C57BL6 mouse exhibits less severe arthritis than the DBA/1 strain [19]. After immunization with collagen type II (CII), inflammatory disease similar to that in arthritis patients is observed. It is accompanied by activation of T and B cells and production of antibodies generated against CII [20].

In this study, we hypothesized that a non-invasive method to quantify bone loss in the CIA mouse model could be accomplished by evaluating reconstructed high quality micro-CT images. We obtained longitudinal images by micro-CT of CIA mice and compared them with images from untreated control mice to quantify the changes in bone volume and density induced by disease. The results of this study demonstrate that micro-CT can be used to quantify bone changes in the CIA mouse model and that micro-CT could be useful as a non-invasive method to monitor the efficacy of new treatments for RA in small animals.

## Methods

### Ethics statement

All animals used in this study received humane care based on guidelines set by the American Veterinary Association as well as in accordance with the *Guide for the Care and Use of Laboratory Animals* (Institute for Laboratory Animal Research, Washington, DC). The experimental protocols involving live animals were reviewed and approved by the Institutional Animal Care and Use Committee of LSU Health Sciences Center at Shreveport. All efforts were made to minimize animal suffering, to reduce the number of animals used and to utilize alternatives to *in vivo* techniques, if available.

### Animals and induction of collagen-induced-arthritis

Nine 12 week old DBA/1 J male mice (Taconic Farms; Hudson, NY) were randomly divided in two groups: a control group of three animals and a CIA group of six animals. The CIA group was immunized with emulsified chicken type II collagen (Chondrex; Redmond, WA). On day 21, the CIA mice were injected with a booster of 100 μg of CII in incomplete Freund’s adjuvant (IFA) by intraperitoneal injection. The control group received no injections.

### Micro-CT imaging

Micro-CT images were acquired on the Tri-Modality FLEX Triumph™ Pre-Clinical Imaging System (GammaMedica-Ideas; Northridge, CA). Each mouse was anesthetized using isoflurane gas. Image sets were acquired at 0, 4, 7, and 9 weeks post-immunization. CT image sets acquisitions lasted 4.27 minutes and utilized beam parameters of 130 μA and 80 kVP. Analyze 10.0 (AnalyzeDirect; Overland Park, KS) was used to perform the image analysis. Regions of interest (ROI) were created for each bone of interest (humerus, femur, and tibia bone). The thresholding utility in Analyze was used to semi-automate the ROI creation (set to 800 Hounsfield units). Each ROI was inspected visually in transverse, coronal, and sagittal slices and edited where appropriate. Finally, the individual ROIs for each bone were compiled together to create 3D representations of the bones of interest. The total number of pixels in the ROI across all slices for a specific bone ROI and mean pixel intensity were obtained and used as measures of bone volume and density. The mean intensity was calculated using volume-weighted normalization. For each bone, the volume-weighted density of each slice was summed and divided by the summed volumes of each slice to obtain the mean density as follows:

$$\text{mean}\;\text{bone}\;\text{density}\;=\;\frac{\sum_{s=1}^{n}\left(\text{slice}\;\text{volume}\;*\text{mean}\;\text{slice}\;\text{density}\right)}{\sum_{s=1}^{n}\left(\text{slice}\;\text{volume}\right)}$$

Each measurement was normalized to a given mouse’s baseline (week zero) scan. Student’s t-tests (unpaired, two-way) were applied to compare control versus CIA mice. Statistical significance was set at P < 0.05.

### Histological analysis

After the final micro-CT imaging session at week 9, the mice were humanely euthanized by CO_2_ asphyxiation. The limb joints were dissected and placed into 4% formalin for one week. The bones were subsequently decalcified by incubation in phosphate buffered saline containing 0.5 M EDTA and 0.5% paraformaldehyde at 4°C for 4 weeks. Afterwards, the joints were paraffin-embedded and sectioned. Joint sections were stained with H&E and mounted on glass slides for histological analysis by light microscopy. Each of the joints was examined by a blinded procedure and classified for the severity of the joint lesions. Four histologic parameters were assessed: inflammation, synovial hyperplasia, pannus formation, and erosion of cartilage/bone, and classified into four grades: grade 0, normal; grade 1, slight; grade 2, moderate; grade 3, severe. A maximum score of 12 was assessed.

## Results

### Analysis of bone density changes by micro-CT

**Figure 1:**
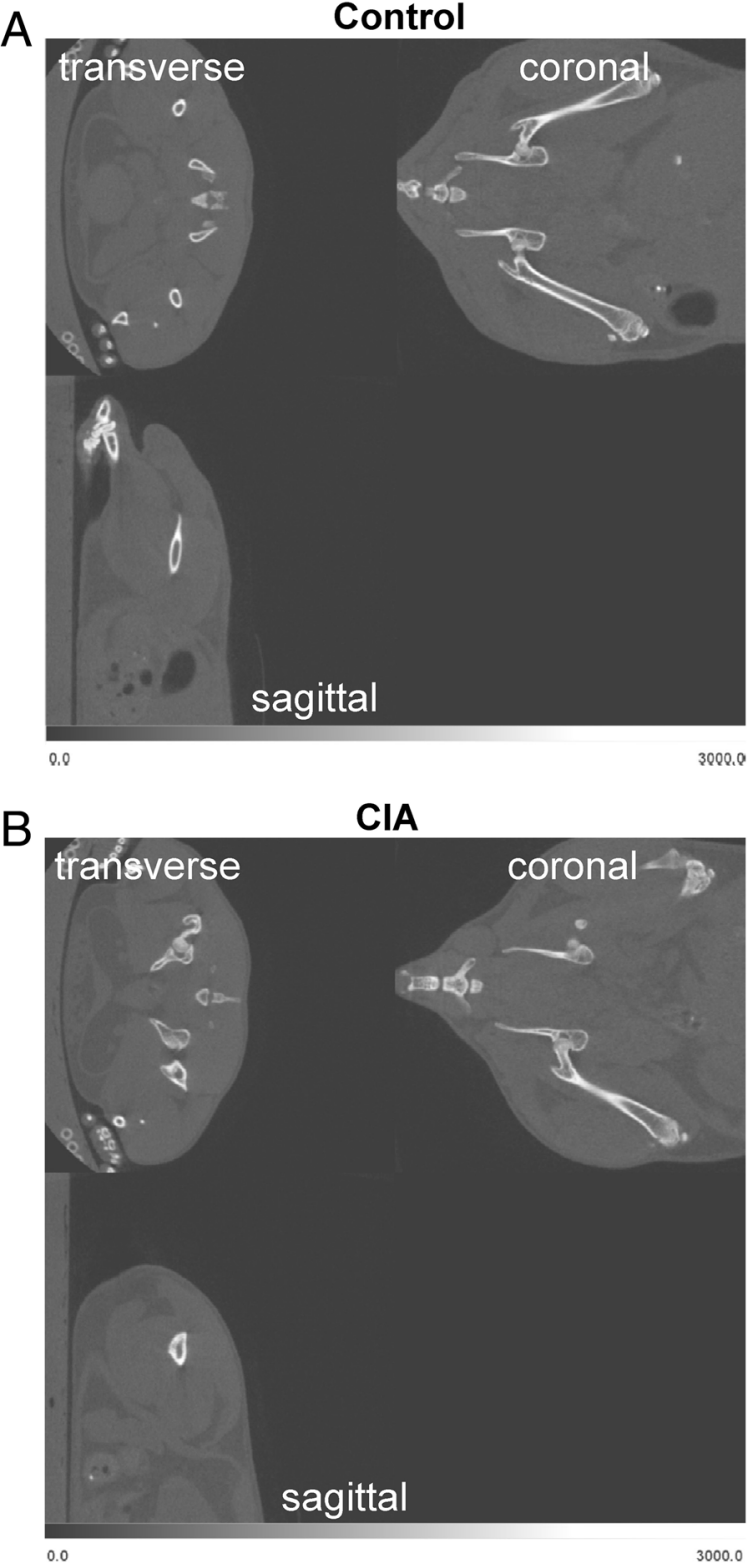
**Visualization of the left femur bone by micro CT.** Whole-body micro-CT images of a representative control **(A)** and CIA mouse at **(B)** week 0. Transverse, coronal, and sagittal planes are shown.

**Figure 2:**
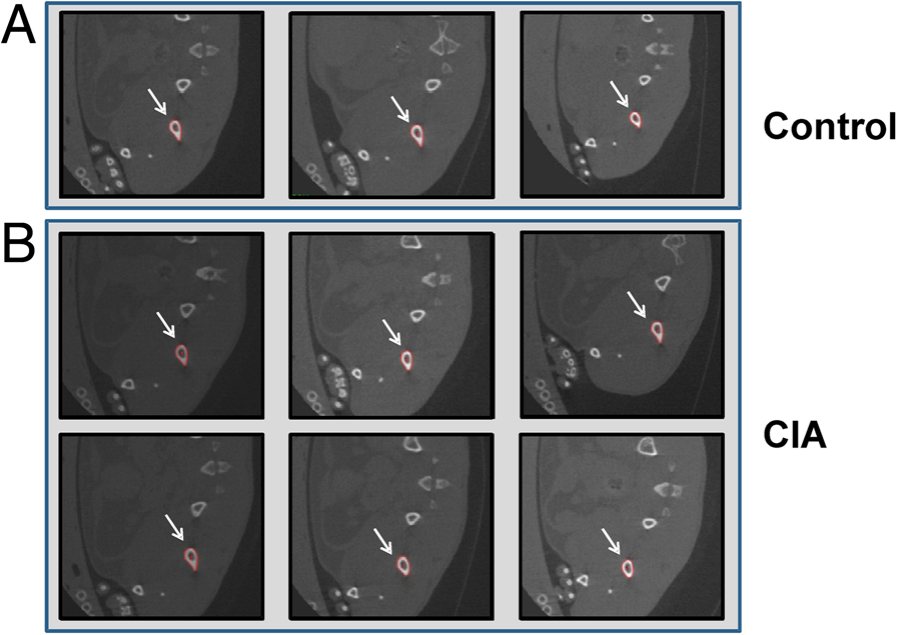
**Measurement of bone density by micro**-**CT.** Segmented slices of femur bones from control **(A)** and CIA mice **(B)** at the first time scan. The arrows indicate regions of interest (ROIs) with the red edges detecting the margins marking the boundaries of the bone.

**Figure 3:**
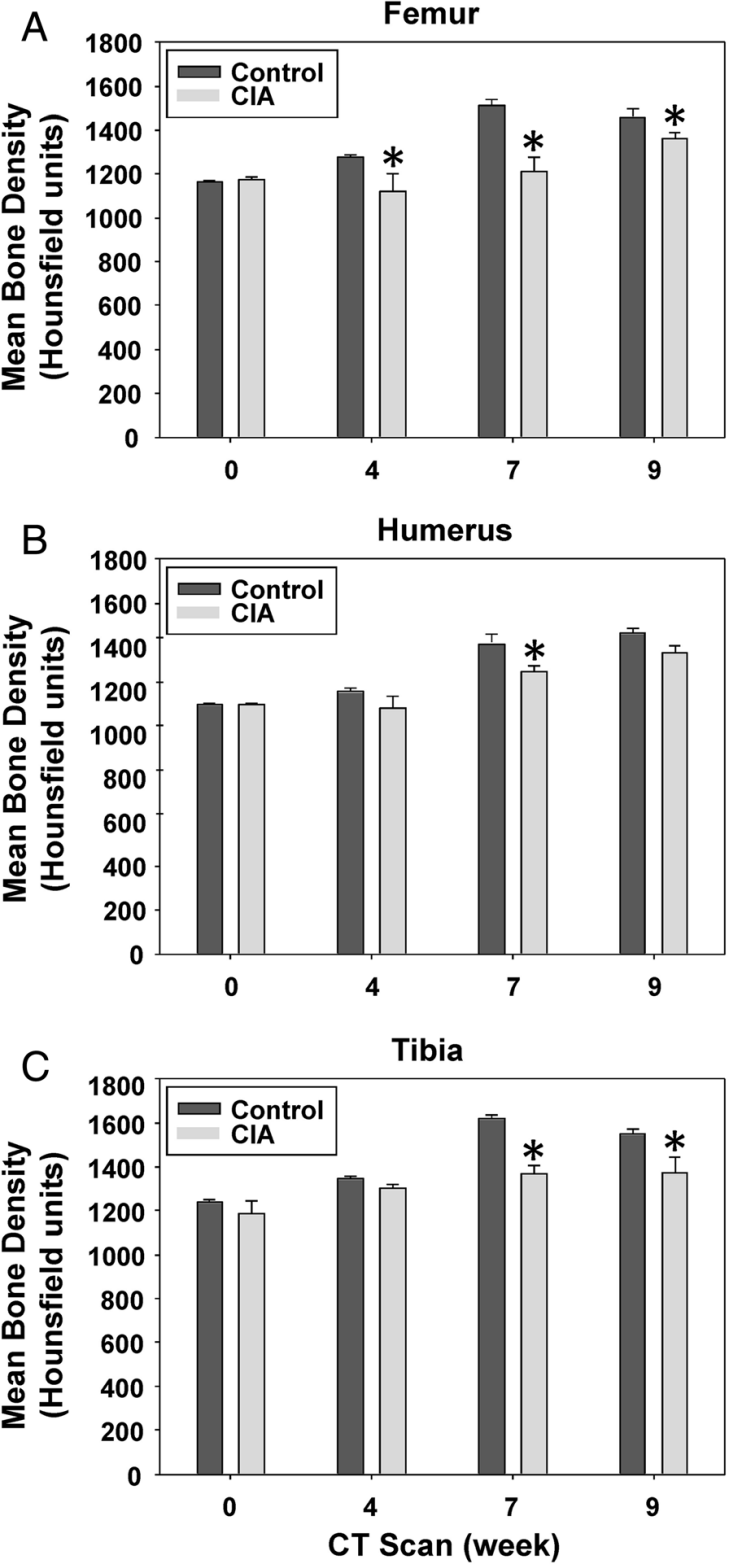
**Mean bone density in the bones of control and CIA mice.** Shown are the mean densities and standard errors over time for **(A)** femur, **(B)** humerus, and **(C)** tibia from control and CIA mouse groups. P values were calculated by comparing each pair of control and CIA densities for each scan time (week 0, week 4, week 7, and week 9), and statistical significance was considered significant (*) if P < 0.05.

All mice were imaged prior to the immunization with CII at week 0 and subsequently when mice developed symptomatic evidence of polyarthritis at weeks 4, 7, and 9. Representative micro-CT images of control and CIA mice at week 0 are shown in Figure 1. Examples of the segmentation CT slices through left femurs of a control and CIA mouse at week 0 are shown in Figure 2. We calculated the mean bone density of femur, humerus, and tibia bones from the CT images. As shown in Figure 3A, the mean bone density of femurs in control mice increased by 25.4% between weeks 0 and 9. In contrast, during the same period the mean bone density of femurs in CIA mice increased only 15.8%. Initial mean bone density between the two groups was not significantly different at week 0, we observed statistically significant differences during subsequent scans at weeks 4, 7, and 9. In a similar fashion, the mean bone density of the humerus bones in the control mice increased by 29% between weeks 0 and 9 (Figure 3B). In contrast, the mean bone density of the humerus bones in the CIA mice increased only 20.9% between weeks 0 and 9; however significant difference was observed only at week 7. Finally, the mean bone density of tibia bones from control mice increased by 25.1% (Figure 3C). In contrast, CIA mice showed only a modest 15.8% increase during the same period that reached statistical significance at weeks 7 and 9.

### Analysis of bone volume changes by micro-CT

**Figure 4:**
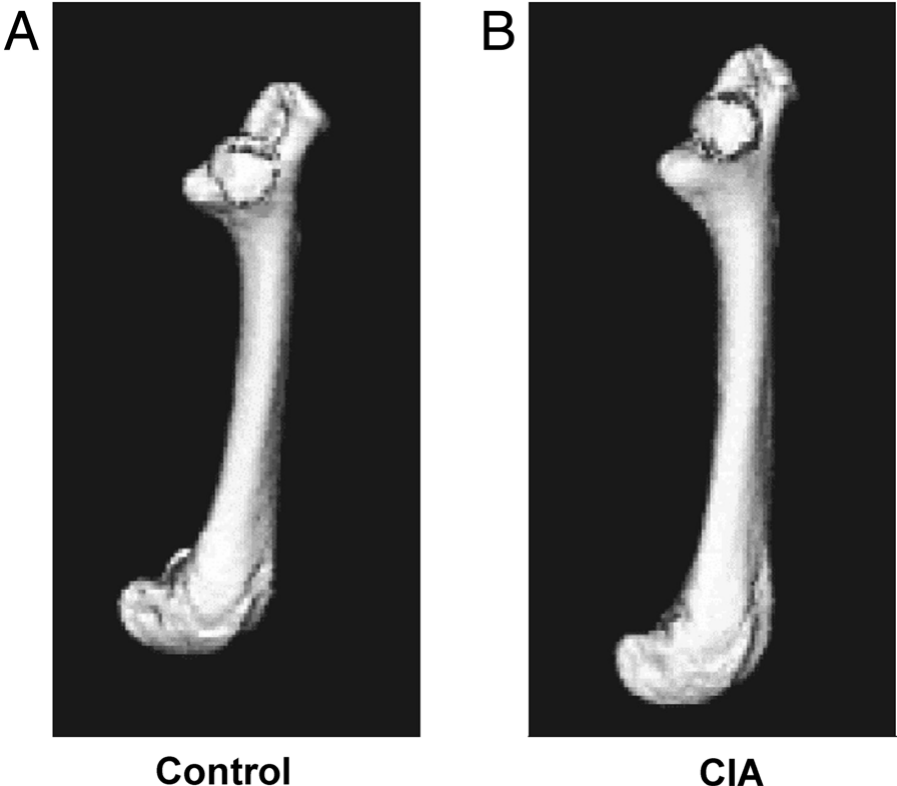
**Measurement of bone density by micro-CT.** Shown are representative longitudinal isosurface volume renderings of the femur from a **(A)** control mouse and a **(B)** CIA mouse from week 0 after initiation of collagen immunization. All rendering thresholds were set to 800 Hounsfield units.

**Figure 5:**
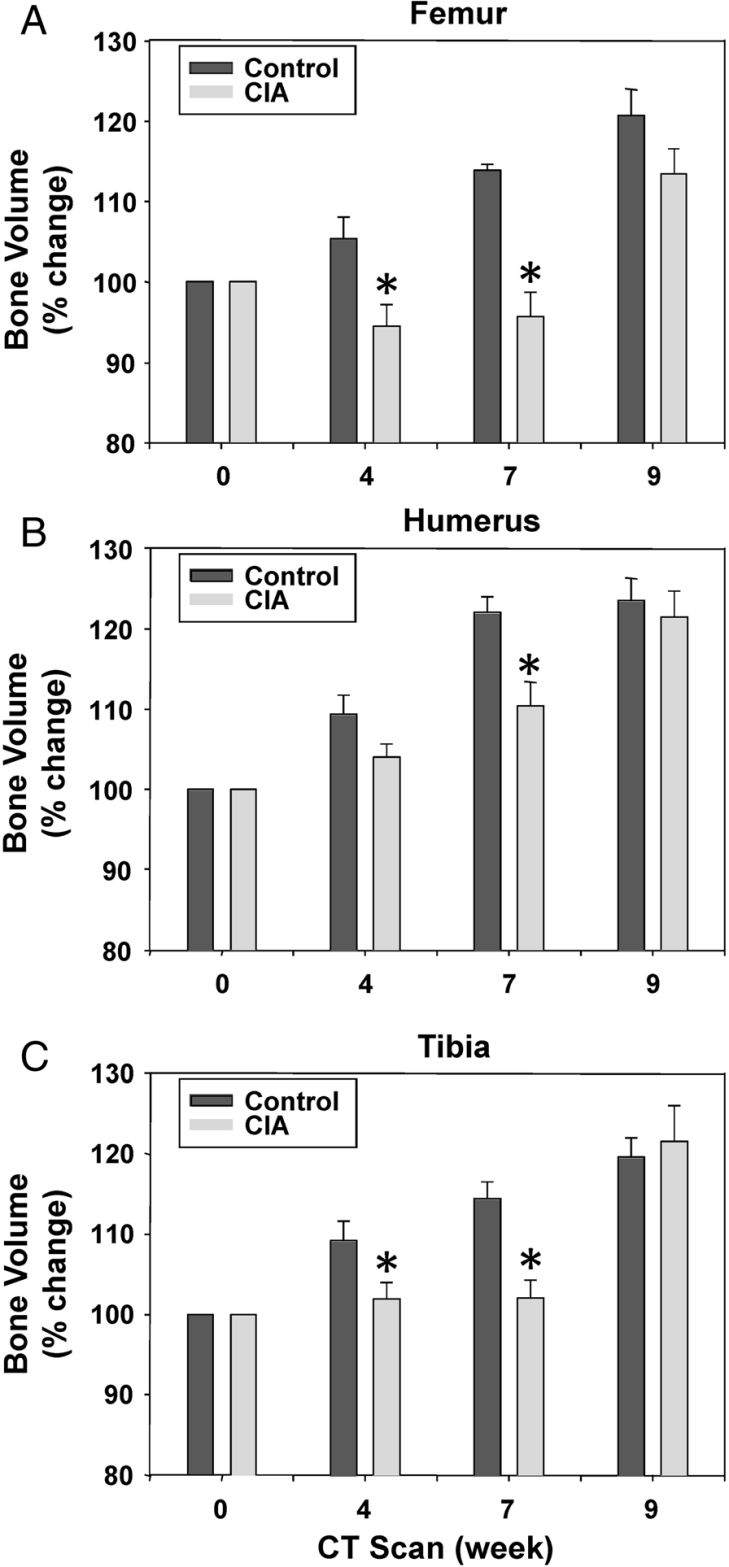
**Mean bone volume in the bones of control and CIA mice.** Shown are the mean volumes and standard errors over time for **(A)** femur, **(B)** humerus, and **(C)** tibia bones from control and CIA mouse groups. P values were calculated by comparing each pair of control and CIA volumes for each scan time (week 0, week 4, week 7, and week 9), and statistical significance was considered significant (*) if P < 0.05.

3D images were created for each left and right bone from humerus, femur, and tibia of control and arthritic mice. Representative left femurs are shown in Figure 4. We then calculated the normalized mean volume from three control and six mice with CIA for each bone analyzed (right and left femurs, right and left humeri, and right and left tibias). To determine whether there were any differences in the mean bone density between volumes of the control and the arthritic mice, we averaged bones from right and left sides. As shown in Figure 5A, the mean bone volume of the femur from the control mice increased by 20.7% from the start of the experiment. In contrast, the mean bone volume of CIA mice increased only 13.5%. Importantly, after the initial mean bone volumes at week 0 (before initiation of immunization) showed no difference, two subsequent scans at weeks 4 and 7 after disease developed showed statistically significant differences. Similar trends were observed when the average bone volume of humeri and tibias were compared between the control and mice with CIA (Figure 5B and C).

### Histological evaluation of disease

Mice with CIA exhibit histological changes that are similar to human patients with RA. Some of the histopathological findings in arthritic mice include joint damage, cartilage and bone erosion, pannus formation, proliferation of the synovium, inflammation, and swelling of the joints [21,22]. In addition, T cell and B cell as well as neutrophil and mast cell infiltration contribute to disease formation [23]. To examine the extent of the disease in our animals, we performed histopathological analysis of bone and tissues after the mice were euthanized at week 9.

**Figure 6:**
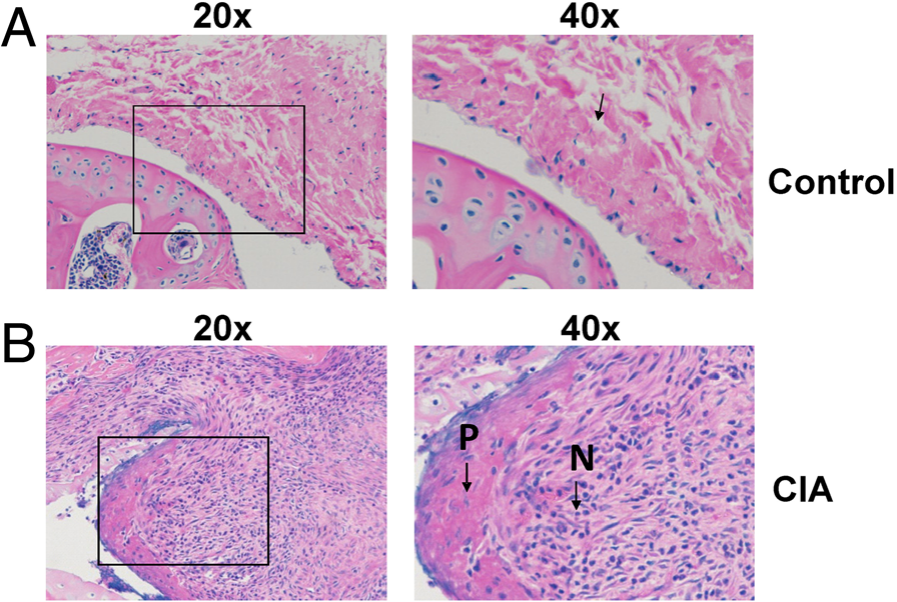
**Comparison of histology in synovial membranes of control and CIA mice.** Shown are representative H&E stained sagittal sections of the humerus and radius-ulna joint examined by light microscopy from **(A)** control and **(B)** CIA mice.

**Figure 7:**
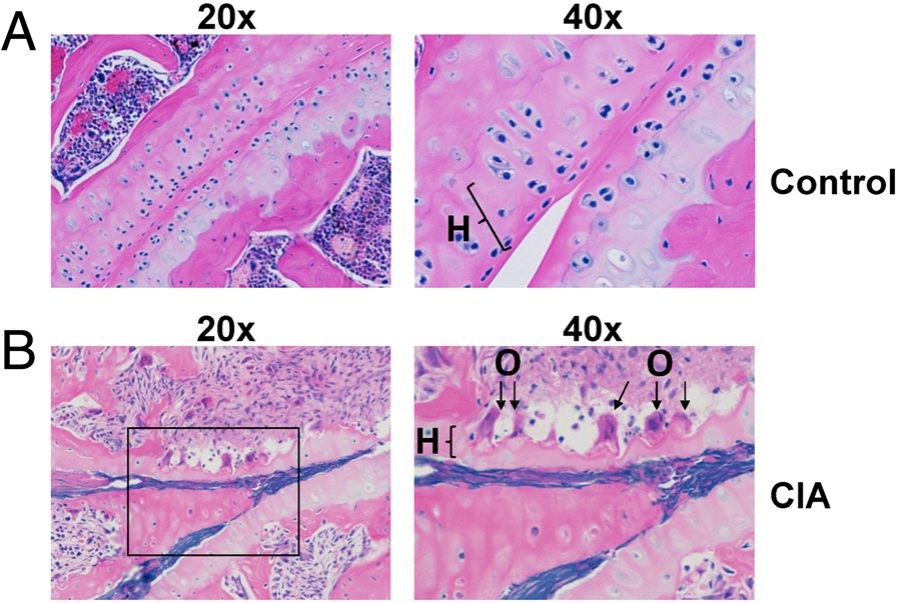
**Comparison of histology in cartilage and bone of control and CIA mice.** Shown are representative H&E stained sagittal sections of the humerus and radius-ulna joint examined by light microscopy from **(A)** control and **(B)** CIA mice.

Representative sections through the humerus and radius-ulna joint of control and CIA mouse are shown in Figures 6 and 7. Soft tissues surrounding the joints of the control mice appeared normal (Figure 6A). We observed that the surfaces of the bones in control mice were covered with smooth hyaline cartilage of the uniform thickness. In contrast, mice with CIA showed severe cartilage and bone and erosion and profuse infiltration of inflammatory cells. Shown in sections representative CIA mice (Figure 6B and Figure 7B), are sagittal sections of the elbow joint.

Representative images from arthritic mice (Figure 6B) revealed thickening of the synovial membrane compared to normal mice (Figure 6A). In RA, the synovium is transformed into a proliferating tissue, called the pannus, which can invade and destroy the underlying joints. We observed the formation of a thick pannus layer (P) in mice with the CIA (6B). We also noted the presence of a large number of neutrophils (N) under the pannus layer (6B). Another important feature we observed in the CIA mice was a large number of lymphocytes present under the hyperplastic synovial membrane, which was absent in the control mice.

Next we focused on the cartilage surface of the joint. In the images from the control mice (Figure 7A), the soft tissues surrounding the joints appeared normal. The surface of the bone was covered with smooth hyaline cartilage and the thickness of the cartilage was uniform. However, compared with the control mice, the CIA mice had significant changes in the cartilage and bone area, including severe cartilage and bone erosion and inflammatory cell infiltration. The hyaline cartilage (H) of the CIA mice (Figure 7B) was degraded and was thinner than in the normal mice (Figure 7A). There were bone erosions present, as well as the appearance of irregular bone trabecula. Significantly, we observed leukocyte infiltration throughout the damaged joints, which is indicative of a severe inflammatory response. Osteoclast cells (O) were clearly observed to be present under the damaged cartilage in the CIA mice (Figure 7B). However, these could not be found in sections from control mice.

In addition, there was bone erosion present as well as irregular bone trabecula.

To further characterize the differences in disease severity between the groups, we performed histological scoring of inflammation, synovial hyperplasia, pannus formation, and erosion of cartilage/bone on two control mice and five CIA mice (Table 1) in a blinded fashion. Each of the joints was examined and classified for the severity of the joint lesions into four grades: grade 0, normal; grade 1, slight; grade 2, moderate; grade 3, severe. Minimal inflammation, synovial hyperplasia, pannus formation, and bone erosions were evident in the control mice (individual histology scores of 0 to 1). However, the CIA mice exhibited moderate to severe inflammation, synovial hyperplasia, pannus formation, and bone erosions (individual histology scores of 2 to 3) in the majority of joints examined.

**Table 1:** The severity of RA histology in tissue sections of control and CIA mice

		**Shoulder joint**		**Elbow joint**		**Knee joint**		**Ankle joint**	
		**L**	**R**	**L**	**R**	**L**	**R**	**L**	**R**
		**Control mice**							
**Mouse 1**	inflammation	0	1	0	0	0	0	0	1
	synovial hyperplasia	1	0	0	0	0	0	1	0
	pannus formation	0	0	0	0	0	0	0	0
	bone/cartilage loss	0	0	0	0	0	0	0	0
	**Score**	**1**	**1**	**0**	**0**	**0**	**0**	**1**	**1**
**Mouse 2**	inflammation	1	0	0	1	0	0	0	0
	synovial hyperplasia	1	1	1	1	1	0	1	0
	pannus formation	1	0	0	0	0	0	0	0
	bone/cartilage loss	1	1	1	1	0	0	0	0
	**Score**	**4**	**2**	**2**	**3**	**1**	**0**	**1**	**0**
**Mouse 3**	nd								
		**CIA mice**							
**Mouse 1**	inflammation	0	2	3	2	2	3	3	3
	synovial hyperplasia	1	3	3	2	3	3	3	3
	pannus formation	0	3	3	2	3	3	3	3
	bone/cartilage loss	0	3	3	2	3	3	3	3
	**Score**	**1**	**11**	**12**	**8**	**11**	**12**	**12**	**12**
**Mouse 2**	inflammation	0	3	1	1	3	3	2	3
	synovial hyperplasia	0	3	2	2	3	3	2	3
	Pannus formation	0	3	2	2	3	3	2	3
	bone/cartilage loss	1	2	1	2	3	3	2	3
	**Score**	**1**	**11**	**6**	**7**	**12**	**12**	**8**	**12**
**Mouse 3**	inflammation	2	3	1	2	2	2	1	2
	synovial hyperplasia	1	3	1	3	3	3	2	3
	pannus formation	2	3	1	3	3	3	1	3
	bone/cartilage loss	2	3	1	3	3	3	1	3
	**Score**	**7**	**12**	**4**	**11**	**11**	**11**	**5**	**11**
**Mouse 4**	inflammation	3	3	3	2	3	2	3	3
	synovial hyperplasia	3	3	3	3	3	3	3	3
	pannus formation	3	3	3	3	3	3	3	3
	bone/cartilage loss	3	3	3	2	3	3	3	3
	**Score**	**12**	**12**	**12**	**10**	**12**	**11**	**12**	**12**
**Mouse 5**	inflammation	3	3	3	1	3	3	2	nd
	synovial hyperplasia	2	3	3	1	3	3	3	nd
	pannus formation	2	3	3	1	3	3	2	nd
	bone/cartilage loss	1	3	2	1	2	3	2	nd
	**Score**	**8**	**12**	**11**	**4**	**11**	**12**	**9**	**nd**
**Mouse 6**	nd								

Scoring for the severity of the joint lesions into four grades was assessed as follows: grade 0, normal; grade 1, slight; grade 2, moderate; grade 3, severe.

nd = not done.

## Discussion

The aim of this study was to find a non-invasive method to assess the bone structure changes in the CIA mouse model. Micro-CT allows for two measurements: mean bone density (as measured from pixel intensities) and mean bone volume (as measured with ROI volumes). Our results show that micro-CT scanning is a viable, efficient and sensitive method to obtain a detailed 3D data about skeletal structures. Analysis of the bone volume and mean bone density changes provided statistically significant results even with a small numbers of animals. Mice with CIA had a significant reduction in bone density and bone volume when compared with control animals, which was verified at necropsy (severe trabecular damage accompanied by cartilage destruction). After week 9, histopathological analysis of the affected joints also showed significant inflammatory infiltrate and formation of pannus, which are two notable histological features of human RA disease.

Bone erosions in mice with CIA have not been previously quantified using 3D reconstruction with micro-CT. Our results revealed reductions in the mean bone density and the mean bone volume of the diseased mice when compared with the control animals. In particular, during the third scanning (week 7), volume and density of all measured bones in the arthritic mice deviated significantly from the corresponding bones in the control mice. The differences became statistically insignificant for most measurements during the fourth period scanning (week 9). Importantly, these results correspond to a visual assessment of disease in this mouse model using a disease index score, which peaked between weeks 4–5 and slowly subsided through week 9 (data not shown).

Many techniques and pharmacologic methods have been attempted for detect and quantify changes in bone caused by chronic inflammatory disease like RA. Histopathologic examination of the joints is often used after necropsy. Other approaches, such as testing biomarkers in serum, urine or tissue [4,24-26], may be useful for researchers, but are limited in their sensitivity and accuracy when monitoring disease progression and morphological changes within the bone. The method described here is sensitive enough to allow analysis of changes in bone structure and has been correlated now as a way to monitor disease progression for the CIA model in mice. In addition, this technique allows the conduct longitudinal studies without sacrificing the animals. There are many potential uses for this technique. For example, it could also be used for monitoring other small animal models with similar bone structure damages.

## Conclusions

Our results suggest the micro-CT approach can provide an accurate, non-invasive, and non-destructive method that could be used to monitor disease progression in the CIA model to determine the efficacy of novel therapeutics.

## Abbreviations

RA: Rheumatoid arthritis; CIA: Collagen-induced arthritis; CII: Collagen type II; CT: Computed tomography; H: Hyaline cartilage; IFA: Incomplete Freund’s adjuvant; MRI: Magnetic resonance imaging; N: Neutrophils; O: Osteoclast cells; P: Pannus layer; ROI: Regions of interest; US: Ultrasound

## Competing interests

The authors declare that they have no competing interests.

## Authors’ contributions

AMH, SJL, DVO, and JMM participated in the experimental design of the experiment. SY, EAO, and JMM performed the image acquisition. SY, EAO, and SIC performed the histological tissue assessment. SY, AMH, EAO, SIC, DVO, SJL, and JMM participated in the experimental data analysis and interpretation. SY, AMH, EAO, SIC, DVO, SJL, and JMM participated in writing and revising the manuscript. All authors read and approved the final manuscript.
